# Effect of a tailored leaflet to promote diabetic retinopathy screening among young adults with type 2 diabetes: a randomised controlled trial

**DOI:** 10.1186/s12886-020-1311-y

**Published:** 2020-03-02

**Authors:** Amelia J. Lake, Jessica L. Hateley-Browne, Gwyneth Rees, Jane Speight

**Affiliations:** 1 School of Psychology, Deakin University, Geelong, VIC 3220 Australia; 2 The Australian Centre for Behavioural Research in Diabetes, Diabetes Victoria, Melbourne, 3000 Australia; 3 Centre for Eye Research Australia, Royal Victorian Eye and Ear Hospital, Melbourne, 3002 Australia; 4 Ophthalmology, Department of Surgery, University of Melbourne, Melbourne, 3010 Australia; 5 AHP Research, Hornchurch, UK

**Keywords:** Retinal screening, Diabetic retinopathy, Behavioural medicine, Young adults, Type 2 diabetes, Randomised controlled trial

## Abstract

**Background:**

Young adults with type 2 diabetes (aged 18–39 years) are at risk of early onset and rapid progression of diabetic retinopathy, the leading cause of blindness and vision loss in working age adults. Early detection via retinal screening can prevent most vision loss, yet screening rates are consistently lower among this priority population than the general diabetes population. We aimed to test the effect of a tailored, evidence-based brief health behaviour change intervention (leaflet) on self-reported screening uptake, and previously identified social cognitive determinants of retinal screening.

**Methods:**

A pragmatic, two-arm randomised controlled trial was conducted from September 2014 to April 2015. Participants were stratified by prior screening uptake (Yes/No) and randomly allocated to intervention (leaflet) or ‘usual care’ control (no leaflet). Primary outcome was self-reported screening uptake four weeks post-intervention for ‘No’ participants who had not previously screened for diabetic retinopathy. Secondary outcome variables were changes in knowledge, attitudes, normative beliefs, intention and behavioural skills for all participants, irrespective of prior screening behaviour. To assess intervention effects on secondary outcome variables, we conducted independent samples t-tests (two-tailed) on pre-post change scores.

**Results:**

129 young adults (26% no prior retinal screen) completed baseline; 101 completed post-intervention. Power to determine effect on the primary outcome was curtailed by low recruitment of individuals with no prior retinal screen and loss to follow-up. Attrition was associated significantly with country of birth, language spoken at home, and marital status. Significant intervention effect was observed for one secondary outcome variable: knowledge of diabetic retinopathy (*p* = .03) with moderate effect (partial eta squared *η*^2^ = .05); no adverse effects were reported. Control group participants received the leaflet at study completion.

**Conclusions:**

This study confirms that a well-designed eye health and retinal screening promotion leaflet can increase knowledge of diabetic retinopathy, an important screening predictor. The study highlights the challenges of conducting ‘real-world’ health behaviour change research with this priority population, providing insights for clinicians and researchers. Strategies to recruit, engage and retain hard-to-reach populations are discussed including nonconventional alternatives to randomised controlled trial designs. Trial registration: ACTRN12614001110673, UTN No.: U1111–1161-9803. Registered 20 October 2014 - retrospectively registered https://www.anzctr.org.au/Trial/Registration/TrialReview.aspx?id=367127.

## Background

The increasing incidence of type 2 diabetes (T2D) in young adults (aged 18–39 years) and associated morbidity and mortality has generated significant concern in recent years [1]. Clinical and population-based studies highlight the aggressive nature of younger-onset T2D, and consequent risk of diabetes-related complications by mid-life [2]. Younger age of T2D onset is an independent risk factor for diabetic retinopathy (DR); the leading cause of vision loss and blindness in working-age adults worldwide [3]. Retinal screening (hereafter ‘screening’) is the proven clinical pathway to early detection of DR and prevention of vision loss [4]. Guidelines recommend screening for DR at T2D diagnosis, repeated periodically thereafter [5–7]. Despite this, there is less take up or initiation of screening (hereafter ‘uptake’) among young adults with T2D when compared with other groups [8, 9]. In Australia, screening rates in this priority population are estimated to be 55%, compared with 78% in the general diabetes population [10, 11]. A range of barriers to retinal screening have been identified among this group [12–14], leading to calls for tailored, age-appropriate intervention [15, 16].

Effective interventions to promote health behaviour change have shared elements: content grounded in evidence, underpinned by theoretical constructs; targeting identified behavioural determinants [17]. Using a co-design approach, our multidisciplinary project team developed an evidence-based and theoretically-grounded screening promotion leaflet, targeting factors previously identified as impacting screening behaviour among young adults with T2D [12, 18, 19]. The leaflet was developed to appropriate literacy standards with the input of the priority population and sector stakeholders and has been acknowledged as an example of best practice [20].

**Fig. 1:**
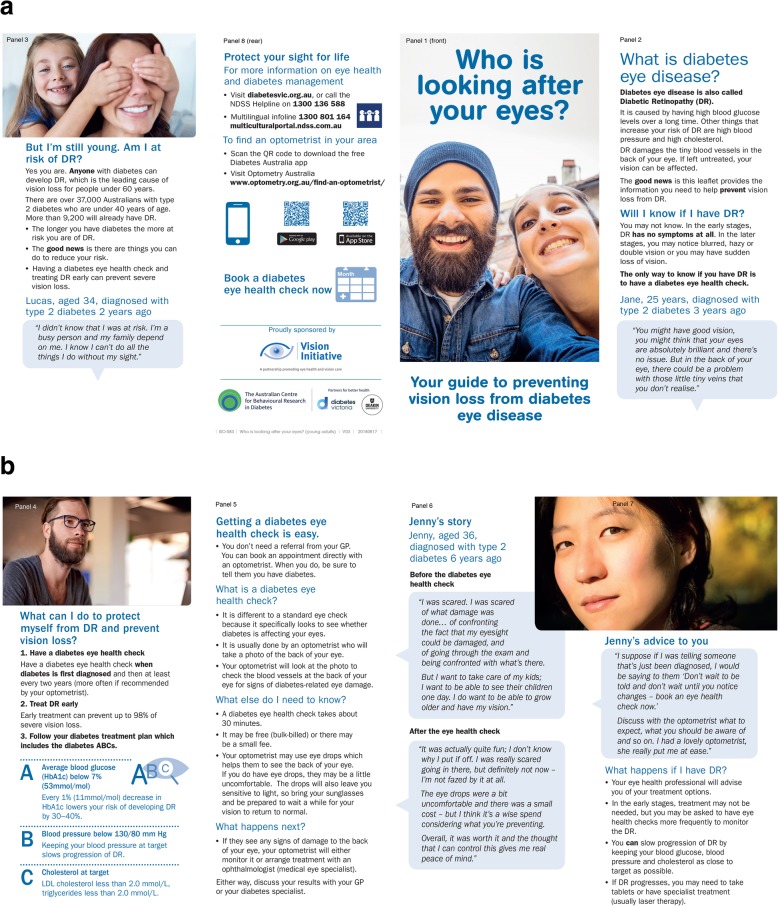
‘Who is looking after your eyes?’ leaflet. ©Vision 2020 Australia 2020, all rights reserved. Panels numbered in typical reading order. Available at: https://bit.ly/2mvJ6yE

The aim of the current study was to test, in a two-arm, parallel-groups randomised controlled trial (RCT), effect of the *Who is looking after your eyes?* leaflet (Figs. 1 a & b). We had two hypotheses: 1) participants who had *not* engaged with DR screening since T2D diagnosis (hereafter ‘unengaged’) and who received the leaflet, would be more likely to initiate screening than unengaged participants who did not receive the leaflet (primary outcome: uptake of screening); 2) all participants who received the leaflet (irrespective of whether they had previously engaged in screening) would show improvement in previously-identified social cognitive determinants of screening behaviour (secondary outcome: change in social cognitive factors).

## Methods

### Study design and randomisation

#### Trial design and registration

A pragmatic, two-arm RCT design was conducted from October 2014 to April 2015. The study was retrospectively registered with Australian New Zealand Clinical Trials Registry, six weeks after recruitment commenced and before randomisation (ACTRN12614001110673, UTN No.: U1111–1161-9803).

#### Sample size calculation and change to trial design after trial commencement

Informed by previous studies [9, 10, 21–23], we anticipated 10% recruitment, 50% baseline screening rate and 40% study attrition. We initially selected a Solomon 4-group design [24] to account for anticipated Question-Behaviour-Effect (QBE), where answering questions about a specific behaviour can influence an individual’s related cognitions, emotions and behaviour [25]. Thus, our initial sample size calculation required 200 unengaged participants (50 per condition in the 4-group design). Calculation used input parameters: effect size of 0.3 [26], 80% power, significance level of 0.05, two-tailed.

However, lower than expected recruitment of unengaged participants in the first few weeks foreshadowed risk of lack of power to detect change in the primary outcome. Consequently, changes were made to trial design (see Additional file 2 for more detail) where: prior to randomisation, the design was modified to a conventional two-arm (intervention/control) RCT, and alternate methods were used to minimise the potential impact of QBE (see Concealment of Study Purpose below). Using the revised design and existing input parameters, 25 unengaged participants in each arm were required to sufficiently power the study to determine effect on the primary outcome.

### Participants

#### Eligibility

Young adults with T2D (18–39 years) registered with Australia’s National Diabetes Services Scheme (NDSS)^1^ were eligible to participate, with registration date used as a proxy for diabetes diagnosis. With approximately 90% of Australians with T2D registered, the NDSS is considered the “best available source to monitor type 2 diabetes in children and young people in Australia” [27]. Exclusion criteria were non-proficiency in English and other diabetes types.

#### Recruitment

Of the approximately 32,000 young adults with T2D registered on the NDSS, 5354 had consented to be contacted for research purposes; all were invited to participate. To protect confidentiality, NDSS staff coordinated study recruitment, including an introductory letter (on NDSS/Diabetes Australia letterhead) and study invitation. Two incentives were offered: a chance to win one of three iPad minis at registration/Stage 1 data collection, AUD$20 upon study completion. A reminder invitation was posted four weeks later and recruitment continued until online study enrolment waned.

### Concealment of study purpose

The purpose of the study was initially concealed to mitigate risk of young adults with T2D who had not engaged in screening declining to participate in a study focused on the behaviour. Consequently, the study invitation advertised the opportunity to participate in a study about ‘diabetes self-management’ with the question about screening status embedded within a suite of items exploring diabetes self-management activities.

### Procedure

Data collection was managed via Qualtrics secure online survey platform (Qualtrics, Provo, UT). Baseline data was collected in two stages to allow identification of participants who had already engaged in screening. Participants were stratified based on engagement with screening and then randomly allocated to ‘leaflet’ intervention or ‘no leaflet’ control. Randomisation sequence was generated by the project manager (AJL) via an online random number generator using a 1:1 ratio [28].

Following a two-month development and piloting process (January to February 2015), the leaflet was posted to all intervention participants in March 2015. Four weeks later, all participants were emailed an invitation to complete a follow-up survey, with survey logic programmed to ensure that previously unengaged participants were asked whether they had engaged in DR screening “since completing the last survey”. The survey also contained all Stage 2 social cognitive items and a fidelity question which asked intervention group participants whether they received the leaflet, and if so, whether they had read it.

Upon completion of the post-intervention survey, all study participants were provided with a transparency statement which explained: the reason for concealment of the true study purpose, why screening is important for all people with diabetes and a link to more information. Control group participants who had been exposed to real-world ‘usual care’ were invited to receive the *Who is looking after your eyes?* leaflet upon provision of their postal address.

### Intervention

Development of the 8-panel, *Who is looking after your eyes?* leaflet (Figs. 1 a and b) is described elsewhere [18].

### Measures

Our survey was reviewed and pilot-tested by stakeholders including young adults with T2D. Baseline data were collected in two stages: i) demographics and clinical characteristics, and ii) social cognitive determinants. At 4-weeks post-intervention, items assessing engagement with diabetes-related health checks (including screening), emotional well-being and all social cognitive determinant items were repeated.

#### Stage 1 Demographic data

Participants provided written consent and demographic data, including gender, age, country of birth, language spoken at home, marital status, level of education, employment status, primary diabetes management, family history of T2D and number of health comorbidities.

Engagement with diabetes-related health checks: was assessed via six separate questions, worded as follows: ‘*Since you were diagnosed with diabetes, have you had your [cholesterol, blood pressure, average blood glucose (HbA1c), kidney function, eye health, feet] checked?’*. A standard definition was provided for each with the aim of minimising reported confusion between standard vision check and screening for DR [29]. Responses to the eye health check component of this question were used to identify: unengaged participants to determine uptake of screening 4-weeks post-intervention (primary outcome).

Depressive symptoms: the Patient Health Questionnaire-2 (PHQ-2) [30], a brief, validated depression screening tool, was included, to identify potential harms arising from the intervention. Responses to PHQ-2 items were summed to produce a total score (range: 0–6), with higher scores indicating more depressive symptoms.

#### Stage 2 Social cognitive determinants

We have previously reported the theory-based development of the 54-item survey used in this study [18]; all items are listed in Additional file 1. In brief, 16 items assessed three knowledge constructs (link between diabetes and vision loss, knowledge of DR, and knowledge of screening). Responses were scored dichotomously (correct / incorrect). Knowledge items were aggregated to form a composite score with higher scores indicating greater knowledge.

Twenty-one items assessed three attitude constructs: i) attitudes to screening, ii) perception of personal risk and iii) anticipated regret at not screening. Three items assessed normative beliefs (such as approval of others and beliefs about the behaviour of similar others) and a further three items assessed intention to screen for DR. For attitudes, normative belief and intention items, responses were scored on either a 5- or 7-point Likert scale, with higher scores representing stronger agreement (items reverse scored where necessary).

Finally, eleven items assessed two behavioural skills constructs: i) perceived control (e.g. ability to seek and attend screening) and ii) overcoming barriers (e.g. ability to identify and address common environmental and psychosocial barriers). Responses were scored on a 5-point Likert scale with higher scores representing greater confidence.

#### Statistical analyses

Data were analysed using the Statistical Package for Social Sciences (SPSS, IBM Corp, Armonk, NY; Ver.23, 2015). To assess factors associated with loss to follow-up, chi-square and independent t-tests (two-tailed) were used to compare baseline demographic characteristics and scores on modifiable behavioural determinants between those who completed and did not complete the study.

Primary outcome: we planned to perform inferential statistical analyses to determine the effect of the intervention on uptake of DR screening. However, insufficient ‘unengaged’ participants provided post-intervention data. As such, the study was underpowered to determine effect of the leaflet on the primary outcome.

Secondary outcome: to assess intervention effects on secondary outcomes, we: i) created change scores by subtracting the baseline composite scores from those at follow-up, ii) conducted independent samples t-tests (two-tailed) on the change scores to assess between-group differences and also conducted paired-samples t-tests to assess within-group changes over time, and iii) calculated effect sizes to determine the relevance of the finding.

Although intention-to-treat and per-protocol analyses were planned, high attrition precluded reliable analysis. Consequently, we elected to exclude cases with missing secondary outcome data pairwise, restricting results to complete cases only for each individual behavioural determinant composite score. Data are presented as means±standard deviation (SD), median (interquartile range, IQR) or n(%). Statistical significance was defined as *p* < 0.05. Effect sizes are described with partial eta squared (*η*^2^, range: 0–1); guidelines for interpretation are: *η*^2^ = 0.01 (small), *η*^2^ = 0.06 (moderate), and *η*^2^ = 0.14 (large effect) [31].

## Results

### Participant flow

**Fig. 2:**
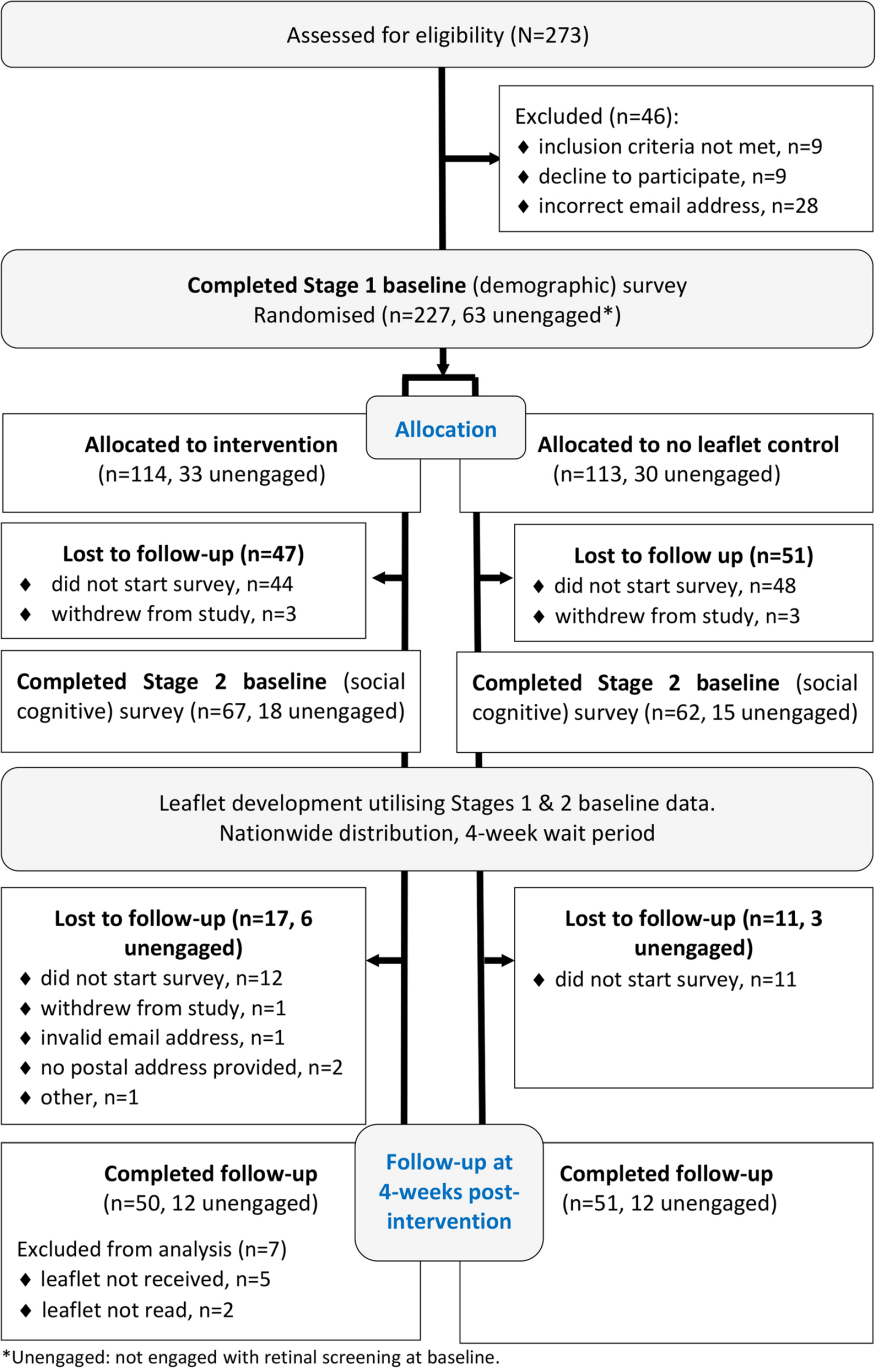
RCT participant flow diagram

Of the 5354 young adults with T2D invited to participate, 273 (5%) visited the study website and completed eligibility screening (see participant flow, Fig. 2). Of those, 227 (83.2%) were eligible, consented to participate and completed the Stage 1 baseline survey (demographic data). At the end of the seven-week recruitment period, 129 (56.8% of the eligible 227 study registrants) completed the Stage 2 baseline survey (social cognitive data). Of those, 101 (78.3%) completed the follow-up survey, 4-weeks later. While there was considerable attrition over the course of the study, there was no evidence of differential attrition between treatment arms (all *p* > .05, data not shown).

Those who completed the study (‘completers’) differed significantly from ‘non-completers’ on three characteristics: compared to non-completers, study completers were significantly more likely to be i) Australian-born (71% vs 48%, *p =* .001), ii) speak English at home (87% vs 71%, *p =* .005), and iii) less likely to be married (61% vs 76%, *p =* .024).

### Baseline characteristics

The average age of the total sample (Stage 1: *N* = 227) was 34 ± 4 years (range: 19–39 years); 56% (*n* = 126) were women, 78% (*n* = 177) spoke English at home, 58% (*n* = 131) were born in Australia and 29% (*n* = 64) were born in Asia. Overall, participants reported short average duration of T2D (1.6 ± 2.5 years), with 66% (*n* = 150) managing their diabetes with oral hypoglycemic agents and 87% (*n* = 197) reporting having engaged with four or more diabetes-related health and complication checks since their diagnosis. Importantly, 72% (*n* = 164) had already engaged with DR screening.

For those who also provided Stage 2 baseline data (*n* = 129), knowledge of an association between diabetes and vision loss was high (1.96 ± 0.20); but lower for knowledge of DR (6.46 ± 2.12) and screening (1.47 ± 0.63). Participants reported high baseline intention to engage in screening (18.45 ± 5.01), strong perceptions of others’ approval (normative beliefs, 13.26 ± 2.12), but only moderate perception of personal risk (12.78 ± 4.38) and anticipated regret at not screening (24.64 ± 6.39). Finally, participants reported moderately positive attitudes to screening at baseline (46.14 ± 6.44), perceived control in attending screening (23.87 ± 5.17) and overcoming barriers (19.57 ± 4.41).

Demographic and social cognitive characteristics of participants who provided both Stage 1 and Stage 2 baseline data are presented by allocated study arm in Table 1.

**Table 1:** Demographic and social cognitive characteristics of study participants who provided both Stage 1 and Stage 2 baseline data, by allocated study arm

	Intervention (*n* = 67)	Control (*n* = 62)
Baseline demographic and clinical characteristics		
Age, years	35.0 (31.0–37.0)	36.0 (33.8–37.3)
Gender: women	40 (60%)	37 (60%)
Country of birth: Australia	41 (61%)	43 (69%)
Main language spoken at home: English	57 (85%)	51 (82%)
Marital status: in a relationship	44 (66%)	39 (63%)
Education level:		
Secondary	13 (20%)	15 (24%)
Trade or certificate	25 (37%)	24 (39%)
Tertiary	29 (43%)	23 (37%)
Employment status: in paid employment	37 (55%)	40 (65%)
Socioeconomic status (SEIFA)^#^	981.7 (73.8)	998.4 (68.3)
Diabetes duration, years	1.53 (2.11)	1.48 (1.78)
Primary diabetes management:		
Lifestyle only	10 (15%)	16 (26%)
Oral medication	44 (66%)	43 (69%)
Insulin	13 (19%)	3 (5%)
Family history of T2D: yes	47 (70%)	47 (76%)
Total diabetes-related health checks (range 0–6)	4.67 (1.31)	4.60 (1.50)
Health comorbidities, number	1.79 (1.33)	1.75 (1.45)
Depressive symptoms (PHQ-2, range 0–6)	2.67 (2.15)	2.15 (1.98)
PRIMARY OUTCOME (engaged with retinal screening since diabetes diagnosis)		
Yes (‘engaged’)	48 (72%)	44 (71%)
No (‘unengaged’)	19 (28%)	18 (29%)
SECONDARY OUTCOME VARIABLES (social cognitive determinants)		
Knowledge		
Knowledge of diabetes/vision link (range 0–2)	1.97 (0.18)	1.94 (0.23)
Knowledge of diabetic retinopathy (range 0–11)	6.28 (2.20)	6.66 (2.03)
Knowledge of retinal screening (range 0–3)	1.46 (0.56)	1.48 (0.70)
Attitudes		
Retinal screening (range 11–55)	46.19 (5.62)	46.12 (7.31)
Risk perception (range 4–28)	13.23 (4.46)	12.26 (4.27)
Anticipated regret (range 6–42)	24.73 (6.19)	24.53 (6.65)
Normative beliefs (range 2–14)	13.41 (1.96)	13.09 (2.29)
Intention (range 3–21)	18.31 (4.78)	18.60 (5.29)
Behavioural skills		
Perceived behavioural control (range 6–30)	23.58 (5.11)	24.20 (5.27)
Overcoming barriers (range 5–55)	19.41 (4.25)	19.75 (4.61)

Data are mean (SD), number (%); age reported as median (IQR)

^#^Socio-Economic Indexes For Areas scores are standardised against a mean of 1000; lower scores indicate disadvantage less than the national average abs.gov.au/websitedbs/censushome.nsf/home/seifa

### Primary and secondary outcomes

Baseline, post-intervention and change scores are presented for all outcome variables by allocated study arm in Table 2.

**Table 2:** Primary and secondary outcomes by allocated study arm and time point

	Time point	Intervention	Control
Primary outcome: Unengaged participants		*n* = 8	*n* = 12
Retinal screening uptake since baseline	Baseline	0	0
	4 weeks	5	3
Secondary outcomes^#^:All participants		*n* = 43	*n* = 51
Knowledge of: Diabetes/vision link (range 0–2)	Baseline	1.97 (.17)	1.96 (.21)
	4 weeks	1.97 (.17)	1.98 (.15)
	Change	0.00 (0.24)	0.02 (0.15)
Diabetic retinopathy (DR) (range 0–11)	Baseline	6.43 (2.38)	6.78 (2.00)
	4 weeks	7.64 (1.97)^b*^	6.86 (2.10)
	Change	1.21 (2.58)^a*^	0.08 (2.07)
Retinal screening (range 0–3)	Baseline	1.45 (.55)	1.42 (.70)
	4 weeks	1.70 (.72)^b*^	1.72 (.70)^b*^
	Change	0.25 (0.78)	0.30 (0.68)
Attitudes: Retinal screening (range 11–55)	Baseline	46.29 (4.99)	46.28 (6.17)
	4 weeks	46.61 (5.70)	45.85 (5.4)
	Change	0.32 (4.36)	−0.43 (4.14)
Risk perception (range 4–28)	Baseline	13.49 (4.24)	12.30 (4.08)
	4 weeks	13.64 (3.91)	12.17 (3.57)
	Change	0.15 (3.18)	−0.13 (3.35)
Anticipated regret (range 6–42)	Baseline	24.62 (5.96)	24.79 (5.55)
	4 weeks	30.67 (5.85)^b*^	29.40 (7.16)^b*^
	Change	6.05 (5.53)	4.62 (5.75)
Normative beliefs (range 2–14)	Baseline	13.26 (2.28)	13.43 (1.44)
	4 weeks	13.10 (2.34)	13.02 (2.66)
	Change	−0.15 (2.38)	−0.40 (2.21)
Intention (range 3–21)	Baseline	18.51 (4.61)	18.72 (5.22)
	4 weeks	18.46 (4.01)	18.83 (4.36)
	Change	−0.05 (4.38)	0.11 (2.12)
Behavioural skills: Perceived control (range 6–30)	Baseline	24.72 (4.10)	24.50 (4.38)
	4 weeks	25.28 (4.24)	24.11 (4.97)
	Change	0.56 (3.48)	−0.39 (4.22)
Overcoming barriers (range 5–25)	Baseline	20.21 (3.27)	19.91 (4.08)
	4 weeks	20.08 (3.43)	19.15 (4.17)
	Change	−0.13 (3.26)	−0.77 (3.44)
Depressive symptoms (PHQ-2, range 0–6)	Baseline	2.38 (2.42)	1.94 (1.96)
	4 weeks	2.05 (2.04)	2.00 (2.01)
	Change	−0.33 (1.78)	0.06 (1.68)

Primary outcome: number of unengaged participants who reported receiving and reading leaflet

Secondary outcomes (all participants): mean (standard deviation); change score = follow-up score minus baseline score (standard deviation); ^#^ Some missing data: range 3–10 dependent upon variable; ^a^ Significant between-condition difference in change scores; ^b^ Significant within-condition difference in change scores; *p < .05

### Screening uptake among unengaged participants

Among the unengaged intervention group participants, there was a trend toward higher screening uptake than among those in the control (no leaflet) group (*n* = 5, 63% and *n* = 3, 25%, respectively). However, insufficient numbers of unengaged participants provided post-intervention data (*n* = 24; 12 in each arm) and the study was under-powered to detect meaningful change on the primary outcome variable.

### Social cognitive determinants

Among all study participants (irrespective of previous screening status), independent-samples t-tests demonstrated no significant between-group differences (all *p* > .05), with the exception of knowledge of DR which increased more among participants in the leaflet intervention arm relative to the control group (M = 1.21, SD = 2.58 and M = 0.08, SD = 2.07, respectively), (t_(72)_ = − 2.213, *p =* .03). The magnitude of the difference in the means was moderate (mean difference = 1.12, 95% CI: − 2.14 to 0.11; partial eta squared = .05).

Participants in both treatment arms reported significant increases in knowledge of screening and anticipated regret (*p* < .05); however, there were no significant between-group difference in change scores.

### Fidelity

The *Who is looking after your eyes?* leaflet was received and read by 43 of the 50 (86%) intervention group participants. Seven participants either did not receive (*n* = 5) or did not read (*n* = 2) the leaflet (with the latter citing ‘lack of time’) and were excluded from outcome analyses. The final post-intervention analysis sample comprised *N* = 94 participants (*n* = 43 intervention; *n* = 51 control).

### Depressive symptoms

PHQ-2 scores were moderate and did not significantly increase over time or differ between groups. No adverse events were reported.

## Discussion

### Summary of findings

To our knowledge, this is the first randomised controlled trial of a tailored intervention designed to engage young adults with T2D with retinal screening. Despite lack of power to assess whether the leaflet increased uptake of screening for unengaged participants, trends in the expected direction were positive. The leaflet was received and read by 86% of the intervention group, demonstrating program fidelity. Overall, however, no firm conclusions can be drawn about the impact of the leaflet on the primary outcome.

The effect of the leaflet on secondary outcomes was promising, with demonstrated increase in knowledge of DR, an important screening enabler [32]. The moderate effect size observed is consistent with those found elsewhere in behavioural medicine [26]. Although the leaflet intervention did not independently impact other identified social cognitive determinants, study involvement was associated with improved knowledge of screening and high anticipated regret, highlighting implications for health policy and practice.

### Limitations

This study has several limitations. Despite rigorous design, careful planning, broad consultation and a nationwide recruitment program, only 4% of eligible NDSS registrants participated. It is likely that low recruitment resulted from a confluence of cohort-specific barriers (e.g. busy life stage, high rates of depression; 21, 23, [33] and context-specific barriers (e.g. study fatigue from concurrent NDSS-supported research programs, personal communication, D. Rae, National Operations Manager NDSS).

The low overall sample size combined with a high baseline screening rate (72%) resulted in a lack of power to detect change in the primary outcome. Although similar to the general diabetes population screening rate in Australia (78%, 11) national and international data suggest that the true younger adult screening rate is closer to 50% [9, 10], indicating that the current study is likely to have experienced recruitment bias. Possible explanations for recruitment bias, which favors high self-reported screening rates, include: self-selection bias and social desirability and recall bias [34]. Despite efforts to conceal the true nature of the study, asking about self-management behaviour from the outset may have elicited a social desirability bias, of which younger people are considered susceptible [26]. Accuracy of self-report is also vulnerable to recall bias, particularly in the light of acknowledged confusion regarding the difference between screening for DR and a standard eye check [29]. Future studies could overcome risk of bias by not only including definition of screening (as was done in the current study) but also corroboration of self-report with clinical record data [35].

Moderate-to-high baseline scores (ceiling effect) for many of the social cognitive factors indicated favorable beliefs and attitudes to screening. Consequently, there was limited potential to detect an intervention effect on secondary outcome variables. Further, as we would expect to see the greatest change in social cognitive determinants in unengaged participants, the low representation of unengaged young adults with T2D may have exacerbated this issue.

Further, the finding that country of birth and language spoken at home were independently related to attrition is important because of the high representation of people of South Asian origin among young adults with T2D [36]. The impact of marital status on attrition is less clearly understood. Making the generalisation that those in marital relationships may be more likely to have children, it is possible that the burden of child-rearing may have influenced ongoing study participation.

### Implications for policy and practice

Young adults with T2D are characterised by high levels of diabetes-specific distress and depressive symptoms, lower diabetes self-efficacy and impaired quality of life, and report feeling shame and negative judgement for having a condition usually associated with older adulthood [37]. The combined consequence of these factors are lack of engagement with support networks and low screening uptake [8, 38]. An age-appropriate resource delivered directly to young adults with T2D may present one of the few opportunities for intervention.

However, as retinal screening determinants are multi-level and multi-factorial, it is likely that even a well-designed leaflet will be insufficient to change behaviour on its own, a conclusion reached by earlier studies into the effect of screening promotion leaflets targeting general practitioners, and other health behaviour change leaflets targeting youth [39, 40]. Instead, resources, such as this are more likely to be of value if implemented as part of a coordinated initiative which utilises registration, recall and reminders to improve rates of screening [32].

Thus, we make three recommendations. First, considering that most interventions to promote screening achieve statistically significant increases in screening rates [32] and that QBE effects have been demonstrated in other socially desirable behaviours [41], we recommend utilisation of QBE as a strategy for policy and practice. The simple act of asking questions about screening may be sufficient to prompt uptake. Second, ‘number of cues’ has been identified as a predictor of uptake of pre-pregnancy care for young adult women with T2D [42]. Similarly, increasing the number of screening cues for young adults with T2D may have the effect of achieving a ‘tipping point’ to prompt action. Again, the relevance, and quality of messaging is a crucial consideration. Finally, the *Who is looking after your eyes?*’ leaflet was, by necessity, print-based in a size that could be posted to participants [18]. In future, we recommend that the messaging and content be used in the digital platforms accessed by this priority population.

### Implications for future research

Considering that participant recruitment was conducted with the support and involvement of leading national eye health and diabetes stakeholders, and that invitations were extended nationwide to over 5000 eligible young adults with T2D utilising the “best available” platform, replication is not a practical option and alternate evaluation designs need to be considered. One solution advanced in biomedical clinical trials involves a consortium approach to simultaneously evaluate medical treatments while sharing the one placebo arm [23, 33]. However, this is not practical for smaller-scale programs.

Instead, emerging literature on non-conventional evaluation designs for small samples offer alternatives which aim to maintain rigor while maximising statistical power for populations of limited sample size [43]. Dependent upon a variety of factors (e.g. time, priority population characteristics, intervention setting and dose), approaches range from mixed-methods, stepped wedge and interrupted time-series, to dynamic waitlisted and regression point displacement designs. However, if a conventional, gold standard RCT design is desired (and a sufficient sample size is achievable), a Solomon 4-group design [24] may be needed to account for the likely presence of QBE.

Finally, our experience suggests that future research programs would need to make concerted efforts from the outset to recruit young adults with T2D from culturally and linguistically diverse communities. Suggestions include: collaboration with community-based organisations and faith centers, language sensitivity and competency, and personal contact [32, 44].

## Conclusion

The aim of this study was to evaluate a tailored, evidence-based leaflet to promote screening uptake among young adults with T2D. To date, there has been a lack of evidence-based development of screening promotion resources broadly, and a complete absence for young adults with T2D, an under-researched, burgeoning priority population. Despite rigorous study design and conduct, and proactive recruitment and retention initiatives, we faced many of the challenges experienced by ‘real-world’ health behaviour change intervention studies conducted with diverse or disadvantaged groups. These included: low recruitment from a small population base, high attrition, and consequent lack of statistical power. Notwithstanding its limitations, this study has demonstrated that a tailored, evidence-based leaflet can improve knowledge of DR among young adults with T2D. Other non-significant trends suggest the leaflet has the potential to be useful for promoting uptake within a broader, nationally-coordinated screening program and via various media.

## Supplementary information

**Additional file 1.** Questionnaire items assessing modifiable social cognitive determinants associated with retinal screening

**Additional file 2.** Changes to Methods after Trial Registration

## Data Availability

The datasets used and/or analysed during the current study are available from the corresponding author on reasonable request.

## Abbreviations

ACTRN: Australian and New Zealand Clinical Trials Registry
DR: Diabetic Retinopathy
DUHREC: Deakin University Human Research Ethics Committee
HbA1c: Glycated Haemoglobin
IQR: Interquartile Range
NDSS: National Diabetes Services Scheme
PHQ: Patient Health Questionnaire
QBE: Question Behaviour Effect
RCT: Randomised Controlled Trial
SD: Standard Deviation
SPSS: Statistical Package for Social Sciences
T2D: Type 2 Diabetes
UTN: Universal Trial Number

## Footnotes

1 The NDSS is an initiative of the Australian Government administered with the assistance of Diabetes Australia. It supports people with diabetes to self-manage their condition by providing subsidised access to diabetes-related products and services.
