# Supplementary material for: Effect of a tailored leaflet to promote diabetic retinopathy screening among young adults with type 2 diabetes: a randomised controlled trial
Source: BMC Ophthalmol. 2020 Mar 2;20:80. doi: 10.1186/s12886-020-1311-y (PMC7053154; doi:10.1186/s12886-020-1311-y)
Supplement: Supplementary file 1 — Additional file 1. Questionnaire items assessing modifiable social cognitive determinants associated with retinal screening [file 12886_2020_1311_MOESM1_ESM.docx]

**Additional File 1**

**Questionnaire items assessing modifiable social cognitive determinants associated with retinal screening**

| VARIABLE | | No. of items | Score range |
| --- | --- | --- | --- |
| Knowledge of… | |  |  |
| i) Diabetes/vision loss link | | 2 | 0-2 |
| Diabetes can lead to vision loss |  |  |  |
| All people with diabetes are at risk of diabetic retinopathy |  |  |  |
| ii) Diabetic retinopathy (DR) | | 11 | 0-11 |
| Prevalence rates of DR amongst people with diabetes |  |  |  |
| DR can cause vision loss or blindness |  |  |  |
| DR can develop without symptoms |  |  |  |
| DR is influenced by high blood pressure |  |  |  |
| DR is influenced by high cholesterol |  |  |  |
| DR is treatable if detected early via an eye health check |  |  |  |
| DR is more likely to develop the longer you have diabetes |  |  |  |
| DR is influenced by high blood glucose |  |  |  |
| Early DR is asymptomatic |  |  |  |
| Recommended target HbA1c^*^ |  |  |  |
| Recommended target blood pressure |  |  |  |
| iii) Screening | | 3 | 0-3 |
| Which health professional would you most likely to see for a DR examination? | | |  |
| When should a person with diabetes have their first eye examination? | | |  |
| Recommended eye examination frequency if no DR present? | | |  |
| Attitudes to… | |  |  |
| 1. Retinal screening^c^ (α=.86)   For me to have an eye health check for DR would... | | 11 | 11-55 |
| ...be a good idea |  |  |  |
| ...(not)^#^ be ‘unpleasant’ |  |  |  |
| ...be wise |  |  |  |
| ...(not)^#^ be ‘difficult’ |  |  |  |
| ...(not)^#^ be ‘frightening’ |  |  |  |
| ...(not)^#^ be ‘unnecessary’ |  |  |  |
| ...be reassuring |  |  |  |
| ...be important |  |  |  |
| ...be beneficial |  |  |  |
| ...be comfortable |  |  |  |
| ...be empowering |  |  |  |
| 1. Risk perception^b^ (α=.70) | | 4 | 4-28 |
| I believe I will develop DR due to my diabetes |  |  |  |
| I expect to be diagnosed with DR at my next eye health check |  |  |  |
| I believe I can reduce my risk of vision problems if I manage my diabetes well | | |  |
| I believe I will develop vision problems due to diabetes |  |  |  |
| 1. Anticipated regret^b^ (α=.87)   If I did NOT have an eye health check for DR, I would feel... | | 6 | 6-42 |
| ...indifferent |  |  |  |
| ...concerned |  |  |  |
| ...fearful |  |  |  |
| ...worried |  |  |  |
| ...regretful |  |  |  |
| ...guilty |  |  |  |
| Normative beliefs^b^ (α=.93, Pearson’s r=.87) | | 2 | 2-14 |
| My family/close friends would approve of me attending an eye health check… | | |  |
| My health professionals would approve of me attending an eye health check… | | |  |
| *Additional normative item:* Most people I know with diabetes  have regular eye health checks^b^ | 1 | 0-7 |  |
| Intentions to…^b^ (α=.98) | | 3 | 3-21 |
| I plan to attend an eye health check… |  |  |  |
| I will make an effort to have an eye health check… |  |  |  |
| I intend to have an eye health check… |  |  |  |
| Behavioral skills… | |  |  |
| 1. Perceived control^d^ (α=.87)   How confident are you that you... | | 6 | 6-30 |
| …know what steps you can take to reduce the risk of developing DR? | | |  |
| …will have regular eye health checks? |  |  |  |
| …know how to make the appointment for an eye check? |  |  |  |
| …will remember to have an eye health check in the next four weeks OR when it  is next due? | | |  |
| …will attend the eye health check that you have booked? |  |  |  |
| …can reschedule the eye health check to a different time or day if needed? | | |  |
| 1. Overcoming barriers^d^ (α=.76)   How confident are you that you… | | 5 | 5-25 |
| …can talk to your doctor about your eye health? |  |  |  |
| …can find the time to attend an eye health check in the next four weeks OR  when it is next due? | | |  |
| …will mention you have diabetes when you make the eye check appointment? | | |  |
| …can resume your normal activities immediately after the eye health check? | | |  |
| …can afford to pay for the eye health check, if there is a charge? | | |  |

DR: diabetic retinopathy.

~Cronbach’s alpha was generated for all motivational and behavioral skills constructs; Pearson’s r generated for 2-item construct. DR: diabetic retinopathy, ^#^responses reverse coded. ^*^Indicator of glycemic control (measure of average blood glucose levels over the past 8-12 weeks). Scoring for individual items: ^a^incorrect / correct; ^b^1 (Strongly disagree) to 7 (Strongly agree), ^c^1 (Strongly disagree) to 5 (Strongly agree), ^d^1 (Not at all confident) to 5 (Extremely confident).
