# Supplementary material for: Effect of a tailored leaflet to promote diabetic retinopathy screening among young adults with type 2 diabetes: a randomised controlled trial
Source: BMC Ophthalmol. 2020 Mar 2;20:80. doi: 10.1186/s12886-020-1311-y (PMC7053154; doi:10.1186/s12886-020-1311-y)
Supplement: Supplementary file 2 — Additional file 2. Changes to Methods after Trial Registration [file 12886_2020_1311_MOESM2_ESM.docx]

**Additional file 2**

**Changes to methods after trial registration.**

Based on experience from an earlier qualitative study [1], the original design for this project addressed a potential source of bias known as question-behaviour effect (QBE) [2]. Common to socially desirable behaviours, QBE is a phenomenon whereby answering questions about a specific behaviour can influence an individual’s cognitions, emotions and subsequent behaviour. QBE has been detected in relation to a range of health-related behaviours, including blood donation, cervical screening, uptake of influenza vaccination and health checks [3-5].

Researchers have highlighted QBE as a source of bias in health behaviour change interventions [6], urging caution in the use of pre-test measures [3]. To account for QBE, we originally selected a Solomon 4-group study design [7], in which participants are assigned at random to a combination of four pre-test/intervention groups in a 2x2 factorial design. In a Solomon 4-group design, half of all participants within each condition receive baseline questionnaires and the other half do not, permitting assessment of both the intervention and the interaction of pre-test items. If pre-test sensitization, or QBE, is present, the effect would be expected to be larger than ‘no pre-test plus intervention’.

Informed by previous intervention studies among young adults with T2D [8, 9], we anticipated low recruitment (10%) [10, 11] estimated a low previous screening rate (50%) [12, 13] and high attrition (40%) [9]. Sample size calculation was computed using input parameter of an effect size of 0.3 [14], which required 200 participants not engaged with screening (50 per condition in the 4-group design) at follow-up for 80% power, using a significance level of 0.05 (two-tailed). To fulfil this requirement, it was estimated that we would need to mail recruitment invitations to 3,400 eligible NDSS registrants. However, subsequent discussions with representatives from the NDSS suggested that recruitment rates would be lower than reported in the literature due to a high number of invitations to concurrent research opportunities being disseminated by the NDSS. Consequently, the planning team agreed to send recruitment invitations to all eligible NDSS registrants who had consented to be contacted for research (N=5,354).

At the end of the seven-week recruitment period, and after an additional reminder invitation was mailed, only 63 young adults who had not screened for DR had registered for the study; less than the required minimum sample size. A senior biostatistician reviewed the study design and advised to replace the Solomon 4-group design with a standard 2-group RCT design (leaflet intervention vs no-leaflet control). A second power analysis confirmed that the effect of the leaflet on the primary outcome could be assessed with a sample size of 50 per condition (including 25 ‘unengaged’ per condition); power and significance levels remained the same.

RCT design modification received Deakin University Human Research Ethics Committee approval in November 2014 and was added to trial registration ACTRN12614001110673.

**References for Supplementary File 2**

1. Lake AJ, Browne JL, Rees G, Speight J. What factors influence uptake of screening among young adults with type 2 diabetes? A qualitative study informed by the Theoretical Domains Framework. *Journal of Diabetes and its Complications* 2017;31(6):997-1006.

2. Spangenberg ER, Sprott DE, Knuff DC, Smith RJ, Obermiller C, Greenwald AG. Process evidence for the question–behavior effect: Influencing socially normative behaviors. *Social Influence* 2012;7(3):211-28.

3. Sandberg T, Conner M. A mere measurement effect for anticipated regret: impacts on cervical screening attendance. *Br J Soc Psychol* 2009;48(Pt 2):221-36.

4. Godin G, Germain M, Conner M, Delage G, Sheeran P. Promoting the Return of Lapsed Blood Donors: A Seven-Arm Randomized Controlled Trial of the Question–Behavior Effect. *Health Psychology* 2013.

5. Conner M, Godin G, Norman P, Sheeran P. Using the question-behavior effect to promote disease prevention behaviors: two randomized controlled trials. *Health Psychology* 2011;30(3):300-9.

6. McCambridge J. From question-behaviour effects in trials to the social psychology of research participation. *Psychology & Health* 2015;30(1):72-84.

7. Solomon RL. An extension of control group design. *Psychological Bulletin* 1949;46(2):14.

8. Zeitler P, Chou HS, Copeland KC, Geffner M. Clinical trials in youth-onset type 2 diabetes: needs, barriers, and options. *Current Diabetes Reports* 2015;15(5):1-8.

9. Nguyen TT, Jayadeva V, Cizza G, Brown RJ, Nandagopal R, Rodriguez LM, et al. Challenging Recruitment of Youth With Type 2 Diabetes Into Clinical Trials. *Journal of Adolescent Health* 2013;54(3):247-54.

10. Browne JL, Nefs G, Pouwer F, Speight J. Depression, anxiety and self-care behaviours of young adults with Type 2 diabetes: Results from the International Diabetes Management and Impact for Long-term Empowerment and Success (MILES) Study. *Diabetic Medicine* 2014;32(1):133-40.

11. Johnson EJ, Niles BL, Mori DL. Targeted recruitment of adults with type 2 diabetes for a physical activity intervention. *Diabetes Spectrum* 2015;28(2):99-105.

12. Diabetes Australia. Young Adults with Diabetes Needs Analysis. Canberra: Diabetes Australia, Canberra.; 2006 [1 June 2017]; Available from: http://static.diabetesaustralia.com.au/s/fileassets/diabetes-australia/6321f173-1642-42ed-8501-e95dc1ffa189.pdf

13. Wang SY, Andrews CA, Gardner TW, Wood M, Singer K, Stein JD. Ophthalmic screening patterns among youths with diabetes enrolled in a large US managed care network. *JAMA Ophthalmology* 2017;35(5):432-8.

14. Johnson BT, Scott-Sheldon LAJ, Carey MP. Meta-synthesis of health behavior change meta-analyses. *American Journal Of Public Health* 2010;100(11):2193-8.
